# Involvement of the skin during bluetongue virus infection and replication in the ruminant host

**DOI:** 10.1186/1297-9716-43-40

**Published:** 2012-04-30

**Authors:** Karin E Darpel, Paul Monaghan, Jennifer Simpson, Eva Veronesi, Harriet W Brooks, Joe Brownlie, Haru-Hisa Takamatsu, Philip S Mellor, Peter PC Mertens

**Affiliations:** 1 Vector-borne Viral Disease programme, Institute for Animal Health, Ash Road, Pirbright GU240NF, United Kingdom; 2 Department of Pathology and Infectious Diseases, Royal Veterinary College, Hawkshead Lane, North Mymms, Hatfield, Herts, AL9 7TA, United Kingdom; 3 Current Address: Australian Animal Health Laboratory, 5 Portarlington Road, Geelong, VIC 3220, Australia; 4 Current Address: Histopathology, Maidstone Hospital, Hermitage Lane, Maidstone, Kent, ME16 9QQ, United Kingdom; 5 Current Address: Columbia University, Center for Infection and Immunity, 722 West 168th Street, New York City, NY, USA; 6 EcoHealth Alliance (formerly Wildlife Trust), 460 West 34th Street, New York City, NY, USA; 7 Current Address: Department of Health, Richmond House, 79 Whitehall, London, SW1A 2NS, United Kingdom

## Abstract

Bluetongue virus (BTV) is a double stranded (ds) RNA virus (genus *Orbivirus*; family *Reoviridae*), which is considered capable of infecting all species of domestic and wild ruminants, although clinical signs are seen mostly in sheep. BTV is arthropod-borne (“arbovirus”) and able to productively infect and replicate in many different cell types of both insects and mammalian hosts. Although the organ and cellular tropism of BTV in ruminants has been the subject of several studies, many aspects of its pathogenesis are still poorly understood, partly because of inherent problems in distinguishing between “virus replication” and “virus presence”.BTV replication and organ tropism were studied in a wide range of infected sheep tissues, by immuno-fluorescence-labeling of non-structural or structural proteins (NS2 or VP7 and core proteins, respectively) using confocal microscopy to distinguish between virus presence and replication. These results are compared to gross and microscopic pathological findings in selected organs from infected sheep. Replication was demonstrated in two major cell types: vascular endothelial cells, and agranular leukocytes which morphologically resemble lymphocytes, monocytes/macrophages and/or dendritic cells. Two organs (the skin and tonsils) were shown to support relatively high levels of BTV replication, although they have not previously been proposed as important replication sites during BTV infection. The high level of BTV replication in the skin is thought to be of major significance for the pathogenesis and transmission of BTV (via biting insects) and a refinement of our current model of BTV pathogenesis is discussed.

## Introduction

Bluetongue (BT) is an infectious but non-contagious haemorrhagic disease of ruminants, caused by bluetongue virus (BTV). *Bluetongue virus* is also the type-species of the genus *Orbivirus* within the family *Reoviridae*[1]. BTV is an icosahedral virus (~80 nm diameter) with a genome composed of 10 linear segments of double-stranded (ds) RNA, that is transmitted between ruminant hosts via “biological” insect vectors of the genus *Culicoides* (biting midges) [2,3]. Early records of haemorrhagic disease in sheep in southern Africa indicate that BT has probably been present in the region for many centuries [4]. Today BTV is distributed throughout the temperate and tropical areas of the world and has recently made significant expansions in its northern distribution, causing major outbreaks of disease in several northern European countries [5-7].

The characteristics of BT pathology, particularly its haemorrhagic nature, provided an early indication that microvascular endothelial cells are a major target for infection [4,8-10]. This was later confirmed for other orbiviruses (including epizootic haemorrhagic disease virus [EHDV] and African horse sickness virus [AHSV]) using techniques that included in-situ PCR, in-situ hybridisation, electron microscopy and immuno-fluorescence microscopy, although immuno-fluorescence often resulted in inadequate detection of viral protein [11-19].

Similarly, initial research on BTV pathogenesis suggested a tropism for lymphatic organs [16,20] and it was subsequently shown that disruption of efferent lymphatic flow from the prescapular lymph nodes delayed detection of systemic viraemia [11]. Several subsets of leukocytes including monocytes, macrophages, neutrophil granulocytes, lymphocytes and dendritic cells, have all been implicated in BTV, EHDV and/or AHSV infection [10,12,16,17,20-23]. However there is still uncertainty regarding the relative importance of different leukocyte subsets during BTV infection, their fate after becoming infected and the validity of in vitro studies in comparison with natural infection in ruminants [21,22,24,25].

A pathogenesis model for BT, outlining the dissemination of the virus within the ruminant host, was originally developed on the basis of time-course infection experiments and the isolation of infectious virus from different organs [10,16,20]. This model suggests that after virus is deposited in the skin, usually by infected adult female *Culicoides*, it is drained directly to the regional lymph nodes where it undergoes a first round of replication. Infected leukocytes are thought to be responsible for this early dissemination within the infected host [20]. From the draining lymph node the virus is subsequently thought to be transported via the efferent lymph to the blood stream and is distributed (as a transient low level viraemia) to other lymphatic tissues and the spleen [10,16,20]. The lymphatic tissues of infected animals always contain large amounts of virus (even in animals intravenously inoculated with BTV). Certain subsets of leukocytes (within the regional lymph nodes) are therefore thought to represent primary cellular targets for the first round of virus replication [11,16,20,26].

The virus also infects endothelial cells in several secondary organs (including the lungs, tongue and lips) leading to the development of a high systemic viraemia. During this stage BTV has been isolated from every organ that was investigated [10,20]. Once systemic viraemia reaches a certain threshold level, adult vector species of *Culicoides* could ingest sufficient BTV during blood feeding, to become infected, leading to onward transmission of the virus [27].

Two problems have hampered refinement of this model and the development of more detailed analysis of BTV infection, replication and spread within the mammalian host. Firstly, most of the techniques employed to detect BTV in ruminants ex vivo (such as PCR and virus isolation) do not distinguish between the “presence” of the virus and its “replication”. Secondly, although immuno-fluorescence-microscopy using antibodies against non-structural and structural proteins could potentially differentiate between “replication” and “presence”, it lacks sensitivity and has previously failed to adequately detect viral proteins.

On the other hand confocal microscopy has more recently been used successfully to detect replication of foot and mouth disease virus (FMDV) in pig-tissue sections, while retaining the morphology of the tissue [28].

In this study similar confocal microscopy and immunolabelling were used to detect specific BTV structural (VP7, Core proteins) and non-structural proteins (NS2) with greater sensitivity in thick tissue sections of sheep. The pathogenesis of BTV was investigated by detecting expression of structural and non-structural viral proteins in specific organs, and comparison to the findings of gross and microscopic pathology studies. The skin of some of the infected sheep was exposed to the feeding of naïve adult *C. sonorensis,* to investigate if repeated insect feeding and the resulting inflammatory response would alter the amount of virus at the biting site.

## Methods

### Animals and virus

Five Dorset Poll sheep (ear tag numbers VH56, VH57, VH58, VH66, VH67) were infected with BTV-2, using reconstituted freeze-dried sheep blood (10^5.75^ EID_50_/mL) (Orbivirus Reference Collection (ORC) sample number: RSA1971/03). The virus had not previously been passaged in tissue culture and specific details of this virus strain are available from the ORC web page [29].

Each animal received 1.5 mL of the inoculum subcutaneously (s.c.) into the left site of the neck and 0.5 mL intradermally (i.d.) into the right inner thigh. Individual animals were exposed to different *C. sonorensis* biting regimes on the inner thigh of the right leg and were euthanized individually by an overdose of pentobarbital for necropsy at 3, 4, 6, 8, or 9 days post infection (dpi) respectively. Individual animals were infected on different dates, in order to allow sufficient time after each necropsy for sample processing.

The animal experiment was carried out in the insect proof isolation units at Pirbright laboratory under project-licence PPL 70/5793. All animals were fed daily and had unlimited access to water. Animals were kept for 5–7 days within the housing facilities to allow acclimatisation before any experiment started. After infection, temperature and clinical signs were recorded on a daily basis. Although not shown in detail, EDTA blood samples were taken from the animals every other day to confirm infection by virus isolation.

To confirm results observed in this longitudinal study with BTV-2, additional tissues were also examined from a sheep and a calf infected with the northern European outbreak strain of BTV-8 (ORC isolate number: NET2006/01) at 8 and 10 dpi [30].

#### *Necropsies and tissue collection*

Necropsies of euthanized animals were carried out in the post mortem facilities at IAH Pirbright. To avoid viral contamination necropsies and collection of tissues were always carried out from “external” to “internal” tissues in the following order: skin (both inner thighs, shaven and cleaned), lips; mandibular lymph nodes, prescapular lymph nodes, tonsils, tongue, inguinal lymph nodes, liver; spleen, lung, bronchial lymph nodes and heart. Between tissue collections disposable forceps and scalpel blades were changed to avoid contamination. Tissue samples from skin, prescapular lymph node, tonsil, tongue and inguinal lymph node were also collected from a BTV-8 infected sheep (8 dpi) and calf (10 dpi) [30] for immunolabelling and confocal microscopy studies only.

For immunohistochemistry and confocal microscopy studies, tissues were fixed in 10 mL of 4% paraformaldehyde solution for three hours while being regularly shaken, then transferred into 10 mL PBS. For histological studies, organ samples were fixed in 5 mL 2% paraformaldehyde for 48 h, transferred into of 5 mL of PBS followed by paraffin wax embedding at the histology department at IAH Compton. Sectioning and haematoxylin-eosin staining were carried out either by the histology departments at IAH, Compton or at the histology laboratory of the Pathology department of the Royal Veterinary College, University of London.

#### *Thick tissue sections*

Paraformaldehyde fixed tissue blocks (approximately 0.5 × 0.5 × 0.5 cm^3^) were stored at 4°C in PBS and could be successfully processed for up to 2 months after collection, before degradation resulted in development of high background staining. All tissues were processed using methods similar to those previously described [28]. Briefly, most tissues were cut into 50–100 μm thick sections (average of 2–5 mm^2^) using a vibrating microtome (Leica VT1000 Leica Microsystems, Milton Keynes, UK). However, this method proved to be unsuitable for skin, which was cut manually using a scalpel. Tissue sections were kept in a watch glass, in cold PBS (for up to 48 h) until staining. The sections were incubated in 0.1% TritonX-100 (Sigma, Dorset, UK) in PBS for 60–75 min, followed by a blocking step with 0.5% BSA-PBS overnight in a moist chamber. Occasionally, to confirm specific staining patterns, selected tissues (skin and tonsil) were incubated in 3% goat or horse serum/0.5% BSA-PBS overnight to increase blocking. The sample was then incubated with the first antibody(s) (diluted to the appropriate concentration in PBS/BSA) for 90–120 min at 37°C, washed 10 times with Ca/Mg free PBS for 2 min each, then incubated with the second - species-specific antibody-conjugate (either Alexa 488 or Alexa 568 (Molecular Probes/Invitrogen, Paisley, UK) diluted 1:200 in PBS/BSA. The tissue sections were washed as previously described and incubated for 30 min in a DAPI-solution (4′6′-diamidino-2-phenylindole (Sigma, UK) 1:10000 made up in dH_2_O). This was followed by 5 washes in ultra pure distilled water. The sections were mounted onto slides with a gene frame (Thermo Scientific (Fisher Scientific), Loughborough, UK) using Vectashield (Vector Laboratories, Peterborough, UK) and cover slips (Agar Scientific, Stansted, Essex UK).

To detect BTV proteins in paraformaldehyde fixed tissue sections, two NS2 and one VP7 specific antibody preparations (Orbivirus antibody (ORAB) -1 or ORAB-53 and ORAB-36, respectively) were used in the BTV-2 longitudinal study, while an additional anti-NS2 antibody (kindly provided by Prof. N.J. MacLachlan, UC Davis, later designated ORAB-268) and an anti-core (ORAB-06) were included for analyses of fixed tissue sections from the BTV-8 infected sheep and calf. Details and concentrations of anti-BTV antibodies used are provided in Table 1. Additionally two sheep cell-surface-marker-specific antibodies, mAb CC15 (anti-bovine WC-1 cross-reacting to sheep; γδ T cell subset specific [31]) and anti-ovine CD45 (mAb 151: [32]), both diluted at 1:100, and an mouse anti-vimentin antibody (diluted 1: 200/Sigma) were used. All primary antibodies were extensively tested prior to this study in cell cultures and sections of tissue from uninfected sheep, using confocal microscopy, to ensure their specific reaction. Control sections were labelled in the absence of primary antibodies. Tissue sections were either labelled with single antibody preparation (to detect only either viral antigen VP7 or NS2) or double labelled for both viral proteins (VP7 and NS2) or a viral protein and a cell-surface-marker-specific antibody.

**Table 1:** Anti-BTV antibodies used for tissue confocal microscopy

**Antigen**	Antibody*	**Species**
Anti-NS2 (raised against bacterial expressed/purified NS2)	ORAB01	polyclonal, rabbit (:1000)
Anti-NS2 (raised against bacterial expressed/purified NS2)	ORAB53	polyclonal, rabbit (1:1600)
Anti-NS2 (based on NS2 mAb [33] kindly provided by N.J.MacLachlan)	ORAB268	mouse ascites (1:1000)
Anti-VP7 (raised against gel purified VP7)	ORAB36	polyclonal, mouse (1:100)
Anti-Core (raised against purified BTV core particles)	ORAB06	polyclonal, guinea pig (1:1000)

*All antibodies are stored in the Orbi Virus Antibody Reference (ORAB) collection of the Vector-borne-viral Diseases Programme at IAH Pirbright.

Images were obtained using a LEICA SP 2 confocal microscope equipped with 405-, 488- and 568-laser excitation (Leica Microsystems UK). Additional differential interference contrast (DIC) images were collected concurrently. Overall, in excess of 1000 sections from all of the different tissues were processed. Tissues which proved of special interest (particularly prescapular and mandibular lymph nodes, tonsil, skin and spleen) were processed repeatedly using different tissue blocks to ensure that fully representative tissue sections were examined for each organ.

### General features of tissue sections examined by confocal microscopy

Tissues were processed as described above. Each tissue section was labelled with one or two anti-BTV antibodies, sometimes cell marker antibodies were also included. The anti-BTV antibodies have an “ORAB” number to refer to the **O**rbi**V**irus**R**esearch**A**ntibodies collection of the Arbovirus research group at IAH Pirbright. Specificity and identification of primary antibodies used are indicated in the images and individual figure legends and detected with species-specific fluorescent conjugate. Nuclei are stained in blue using 4′6′-diamidino-2-phenylindole solution (DAPI). Viral proteins are stained either green or red as indicated in images and a merged picture (coincident staining = yellow) is provided, which shows the overlay of staining in one image. Tissue morphology is shown by differential interference contrast (DIC). The magnification of each picture taken is indicated by the scale bars on the images.

### Feeding *C. Sonorensis* on sheep skin

Approximately 250, 3–4 days old, adult female *C. sonorensis,* obtained from the insectaries at IAH Pirbright laboratory, were fed for 30–40 min, in a waxed card pillbox, attached to the inner right thigh of experimental animals. Each animal was exposed to feeding of *C. sonorensis* on the day of infection with BTV-2 and 1 to 2 days afterwards. *C. sonorensis* were also fed on Sheep VH67 (day 4 pi and VH 58 (6 dpi) on the 2 days prior to infection for skin sensitization. Additionally all sheep, with the exception of sheep VH67 (day 4 pi) were exposed to *C. sonorensis* feeding one day prior and on the day of euthanasia so that skin samples from these sheep were collected within 1 h of insect feeding.

## Results

### Clinical disease

No clinical signs of BTV infection were observed in sheep euthanized at 3 or 4 dpi (animals VH56 and VH67 respectively). At 6 dpi, sheep VH58 had hyperaemic nasal and labial mucosa. The animal also exhibited laboured breathing. No other clinical signs or pyrexia were recorded.

By 8 dpi, sheep VH57 had developed a body temperature of 40°C and its face was severely oedemic. The conjunctivae were reddened (bloodshot) and there was intense hyperaemia of the nasal, buccal and gum mucosa, which included small petechial haemorrhages on the gingiva. The animal was in severe respiratory distress and “fluid” sounds were heard by lung auscultation. The coronary bands were hot to the touch and were hyperaemic.

Sheep VH66 was originally assigned for post mortem at 10 dpi however, due to the severity of clinical signs, the animal was euthanized on ethical grounds at 9 dpi. The sheep had developed pyrexia from 7 dpi onwards, with a maximum temperature of 41.5°C at 7 dpi. All of the mucosal surfaces, including the conjunctiva, were extremely hyperaemic and the nasal mucosa was ulcerated and crusted. Severe facial oedema had developed and the animal was in severe respiratory distress, leading to tachypnoea. Coronary bands appeared markedly inflamed on all 4 feet and the animal showed lameness and a disinclination to move.

Clinical disease and pathology of the additional sheep and a calf infected with BTV-8 have been described elsewhere [30].

### Gross pathology and histopathology

No significant gross pathological changes were observed during the necropsy of the sheep euthanized at 3 dpi (VH56). Minor pathological changes were observed in the sheep euthanized on day 4 pi (VH67) where the lymph nodes of the head and neck only (left and right prescapular and mandibular lymph node) were noticeably enlarged.

More pronounced pathological findings were recorded for the sheep at 6 dpi (VH58). The blood vessels on the subcutaneous aspect (under side) of the reflected skin were engorged. The prescapular lymph nodes were enlarged, although the mandibular lymph nodes appeared normal. Petechial haemorrhages were observed on the root of the tongue. The lungs were generally enlarged showing interstitial oedema. The airways (trachea and bronchi) contained sero-sanguineous fluid or froth.

**Figure 1:**
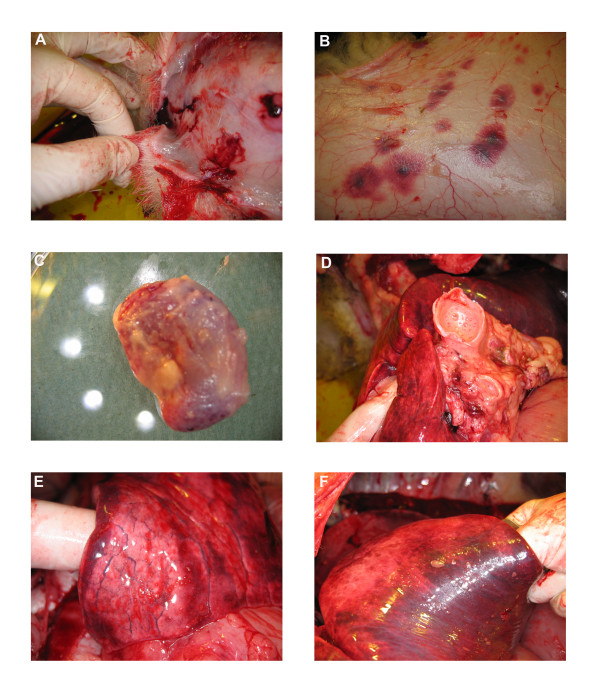
**Gross pathological findings observed in a BTV-2 infected sheep (VH57) at 8 dpi.** Pathological findings observed in a sheep infected with BTV-2 (RSA1971/03) during the necropsy at 8 dpi included severe oedema in the subcutaneous layer of the facial skin **(A)** and severe haemorrhages on the subcutaneous aspect of the skin **(B)**. Both prescapular lymph nodes were markedly enlarged and haemorrhagic **(C)**. All airways (trachea and bronchus) contained serous fluid or froth **(D)** and interstitial oedema was observed in the lung tissue **(E-F).**

At 8 dpi the sheep (VH 57) showed severe sub-cutaneous oedema of the face and lips and the hypodermal layer of the face was gelatinous at necropsy (Figure 1a). Blood vessels, inspected from the subcutaneous side of the skin, were severely engorged, especially in the area exposed to the biting of adult *C. sonorensis*. In addition ecchymotic haemorrhages were seen on the subcutaneous aspect (under side) of the skin all over the animal (Figure 1b). Both prescapular lymph nodes were markedly enlarged and haemorrhagic (Figure 1c). The mandibular, inguinal and bronchial lymph nodes were also enlarged and mild petechiation was observed in the mandibular lymph nodes. The tonsils were enlarged and showed petechial haemorrhages. Mild petechiation was also detected on the root of the tongue and on the heart muscle. On opening the thorax abundant serous fluid was present in the thorax and pericardium. The lungs did not collapse when the thoracic cavity was opened and excised lung tissue immediately sank when placed into paraformaldehyde. Interstitial oedema was observed in the lungs and the airways contained serous fluid or froth (Figure 1d-f).

At 9 dpi marked pathological changes were seen in sheep VH66. Petechiae were present on the mucosal surfaces of the mouth including the tongue. The subcutaneous side of the skin showed the same generalised haemorrhages as described for animal VH57 (at 8 dpi). Prominent haemorrhages were found in all of the excised lymph nodes (prescapular, mandibular, inguinal and bronchial) and the tonsils. The pathological findings of the lungs and thoracic cavity mirrored those described for VH57 (8 dpi), although the heart showed more obvious petechial haemorrhages. The visceral pleura of the lungs had started to adhere to the thoracic pleura of the ribs. Within the abdomen all of the abdominal organs were adhered to each other by firm fibrous tissue. However, the organisation of these adhesions in thorax and abdomen indicate an older cause than the BTV infection.

**Figure 2:**
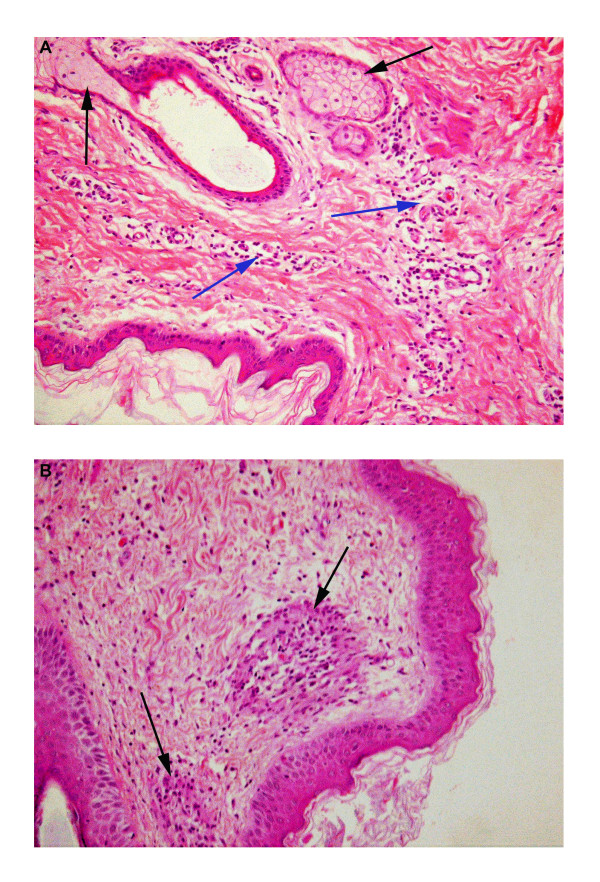
**Histology of the skin of BTV-2 infected sheep exposed to *C. sonorensis *feeding.** Panel **A:** Skin from the BTV-2 infected sheep at 6 dpi (VH58) shows two sebaceous glands, communicating with a hair follicle (black arrows). Clear spaces around capillaries indicate mild dermal oedema. Additionally there is moderate pericapillary aggregation of plasma cells and lymphocytes (blue arrows). Similar changes were seen in skin sections from BTV infected sheep not exposed to *C.sonorensis* feeding, suggesting these histological features are most likely a result of BTV infection (H and E; magnification × 20). Panel **B:** Two granulomas (black arrows) with central neutrophils and eosinophils can be observed in the superficial dermis of the skin at 8 dpi (VH57), most likely as reaction to *C. sonorensis* feeding as they were not observed in sections from unexposed skin areas. Additionally mild oedema in the dermis and mild to moderate aggregation of plasma cells and lymphocytes are recognised (H and E; magnification × 20).

Four tissues (skin, tonsil, lips and lung) were selected from the 5 sheep infected with BTV-2 for histological examination based on their gross pathology and the histological findings are summarised in Table 2. The histological responses of the skin to BTV infection and *C. sonorensis* blood feeding are also shown in Figure 2.

**Table 2:** Summary of histopathological findings in selected tissues of sheep infected with BTV-2 (RSA1973/03)

**Animal**	Skin non-midge fed**	Skin midge fed***	**Tonsil**	**Lips**	**Lung**
**Sheep VH56/ 3 dpi**	Minor engorgement, with indistinct vacuolation of epidermal cells.	Mildly oedematous/focal epidermal crusts/mild dermal inflammation *(lymphocytes, plasma cells and eosinophils).	Epithelial cells markedly vacuolated. Low numbers of eosinophils in tonsil itself.	Oedematous (especially around capillaries) and inflamed* (lymphocytes, plasma cells and eosinophils). Indistinct vacuolation of lip epidermal cells.	Peribronchial connective tissue is markedly oedematous and moderately inflamed*(lymphocytes, plasma cells and eosinophils).
**Sheep VH67/ 4 dpi**	Moderate oedema around capillaries and mild engorgement, mild aggregation of plasma cells around capillaries.	Moderately oedematous, especially around capillaries, with mild engorgement. Mild aggregation of plasma cells around capillaries, low numbers of neutrophils and eosinophils. Focal collagen necrosis. Note: *C. sonorensis* were last fed on this sheep 48 h prior to collection of skin samples.	Epithelial cells markedly vacuolated. Moderate numbers of eosinophils in connective tissue just below epithelium.	Focally dense aggregates of lymphocytes and plasma cells especially around capillaries and adnexae in mid dermis, lighter inflammation in superficial dermis.	Moderate inflammation *(lymphocytes, plasma cells) of peribronchial connective tissue.
**Sheep VH58/ 6 dpi**	Mild oedema and moderate engorgement, sparse dermal inflammation *(neutrophils, lymphocytes, plasma cells)	Moderately oedematous and engorged, with focal degeneration of epidermis. Moderate to marked diffuse dermal inflammation (Figure 2a) *(lymphocytes, plasma cells, eosinophils), with scattered foci of, predominantly, eosinophils associated with collagen necrosis.	Epithelial cells vacuolated. Glassy appearance to nuclei of macrophages, lymphocytes, plasma cells in lymphoid parenchyma.	Oedematous (especially around capillaries) and inflamed *(lymphocytes, plasma cells, eosinophils) – focally markedly so. Indistinct vacuolation of epidermal cells, but also apoptosis, epidermal oedema and erosion.	Peribronchial connective tissue is moderately oedematous and moderately inflamed *(plasma cells, lymphocytes).
**Sheep VH57/ 8 dpi**	Moderate oedema with mild perivascular inflammation *(lymphocytes and plasma cells).	Markedly oedematous (dermis and epidermis). Moderate to marked diffuse dermal inflammation around capillaries *(plasma cells, lymphocytes, eosinophils, neutrophils). Some crusts of surface and dermal foci of macrophages, plasma cells, lymphocytes and eosinophils, including granulomas in superficial dermis (neutrophils and eosinophils centrally with encircling epithelioid macrophages (Figure 2b).	Epithelial cells moderately to markedly vacuolated, numerous lymphocytes and plasma cells in epidermis. Glassy appearance to nuclei of macrophages, lymphocytes and plasma cells in lymphoid parenchyma.	Moderately oedematous (especially around capillaries) and moderately inflamed *(plasma cells, lymphocytes, rarely eosinophils).	Peribronchial connective tissue moderately oedematous and moderately inflamed *(plasma cells, lymphocytes). Diffuse engorgement and congestion.
**Sheep VH66/ 9 dpi**	Minor vacuolation of deep sweat gland cells as well as mild oedema with mild perivascular inflammation *(lymphocytes and plasma cells).	Moderate perivascular oedema and engorgement with patchy haemorrhage. One capillary contained a fibrin thrombus. Moderate to marked inflammation (plasma cells, lymphocytes, eosinophils, and neutrophils).	Areas of markedly vacuolated epithelial cells. There are also areas containing large numbers of lymphocytes and plasma cells in the tonsillar epithelium.	Engorged, haemorrhagic and oedematous (especially around capillaries) with mild inflammation *(plasma cells, lymphocytes, eosinophils and macrophages). Endothelial cells in the walls of some capillaries are degenerate.	Modest peribronchial inflammation *(plasma cells lymphocytes). Mild oedema, moderate engorgement, small numbers of vessels contain fibrin thrombi.

* The order in which the leukocyte subsets are described correlates to their relative abundance.

** Skin collected from the site of the inner thigh not fed upon by *Culicoides*.

*** Skin collected from the site of the inner thigh immediately after it was fed upon by *C. sonorensis*.

### Confocal microscopy and immuno-fluoresence of the BTV-2 longitudinal infections study

#### *Prescapular lymph nodes*

**Figure 3:**
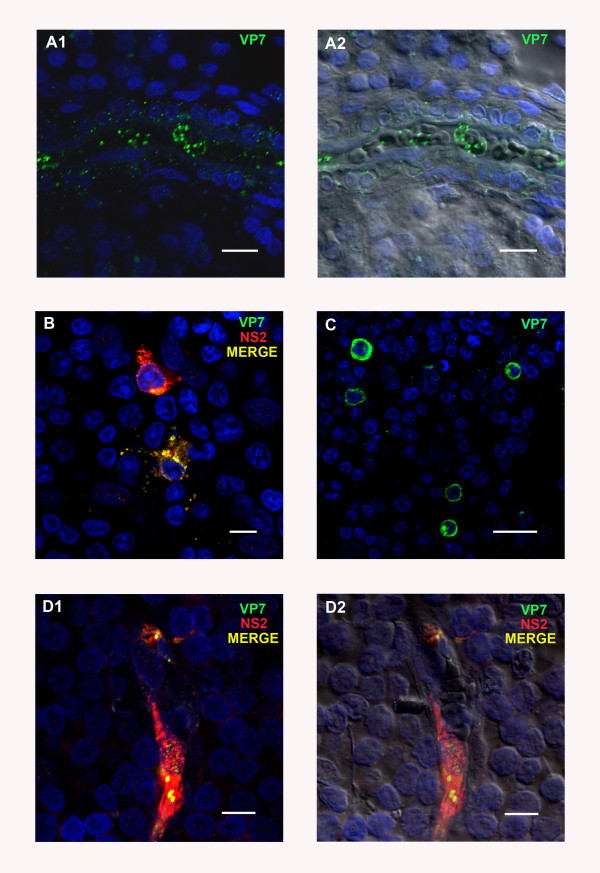
**BTV viral proteins in tissue sections of different sheep lymph nodes at different time points post infection (pi) with BTV-2.A1/A2:** BTV protein VP7 (ORAB36- green) in the lumen of a small blood vessel in the right prescapular lymph node (PLN) at 3 days post infection (dpi). Scale bar: 80 μm. **B** and **C:** Examples of the morphology and distribution of leukocytes positive for BTV proteins VP7 (ORAB36-green) and NS2 (ORAB53-red) in the PLN at 8 dpi. Scale bars: **B**: 8 μm **C:** 20 μm. **D1/D2:** BTV proteins NS2 (ORAB53-red) and VP7 (ORAB36-green) in the endothelium of a capillary in the mandibular lymph node at 8 dpi. Scale bar: 8 μm.

The left and right prescapular lymph nodes were investigated separately, as the left prescapular lymph node represents the “immediate” draining lymph node from the site of inoculation in the neck. BTV proteins, VP7 and NS2 were detected repeatedly, as early as 3 dpi, in leukocytes (as indicated by morphology and CD45 positive labelling), or associated with vascular capillaries, in sections of both prescapular lymph nodes. Both viral proteins were detected within the vascular capillaries, in endothelial cells (as indicated by differential interference contrast (DIC)). VP7 in particular appeared to be located around erythrocytes within the lumen of the blood vessels themselves (Figure 3 a1/a2).

At 3 and 4 dpi, 1 to 10 infected leukocytes were detected per tissue section, increasing to 5–20 infected cells per section, at 6 and 8 dpi. The morphology of infected leukocytes resembled that of lymphocytes, dendritic cells and monocytes (based on the appearance of their cytoplasm, being mononuclear, and their nucleus-cytoplasm ratio) (Figure 3b and c). At 6 and 8 dpi the positively stained leukocytes were often detected within the same area rather than being evenly distributed throughout the lymph node sections. At the peak of infection (6 and 8 dpi), 2–4 areas of leukocytes each containing between 5–10 viral protein positive cells were seen per section.

A much larger number of vascular capillaries were also positively stained for BTV proteins at these later time points (6 and 8 dpi). The infected capillaries were also often seen in close proximity of each other. Overall the numbers of infected cells (endothelial cells and leukocytes) additionally varied markedly between different sections from the same tissue sample (e.g. two different sections at 8 dpi contained 3, or 10 positive/infected cells respectively). Similar variations in the numbers of infected cells (endothelial cells and leukocytes) in different sections from the same tissue sample were detected in all of the organs investigated.

Occasionally both viral proteins were detected in the endothelium of vascular structures within the prescapular lymph nodes, although they could not be conclusively identified as either lymphatic or blood vessels. Fewer infected capillaries and leukocytes were seen in the prescapular lymph nodes of sheep VH66 euthanized at 9 dpi (as compared to 6–8 dpi). Overall, the pattern of leukocyte and capillary infection in the two prescapular lymph nodes was similar, however, throughout the time-course of the experiment, approximately twice as many BTV positive leukocytes and capillaries, were detected in the left lymph node (the side of the infection site) as compared to the right lymph node.

#### *Mandibular lymph nodes*

Overall, similar numbers of cells containing BTV proteins were detected in the mandibular and prescapular lymph nodes. Leukocytes and capillaries (mostly endothelial cells) contained both BTV proteins from 3 dpi onwards. However the numbers of positively stained leukocytes and micro-vascular endothelial cells had increased markedly by 6 and 8 dpi, but were lower again at 9 dpi (Figure 3 d1/d2). At 6 and 8 dpi, more endothelial cells were infected in the mandibular lymph node than in the prescapular lymph nodes. Variations in the numbers of infected cells within and between tissue sections (as described for the prescapular lymph node) were also seen in the mandibular lymph node. In some sections endothelial cells of the larger blood vessels also contained both viral proteins at 6 and 8 dpi.

#### *Inguinal lymph nodes*

At 3 and 4 dpi, BTV proteins were only found in single leukocytes, in some, but not all of the sections from the inguinal lymph nodes. More infected leukocytes were present 6 dpi and infected capillaries were also identified at this time. No further change in the number of positive cells was detected on 8 or 9 dpi. Overall the numbers of infected endothelial cells and leukocytes found were much lower (about 1/10) than in the prescapular and mandibular lymph nodes, despite all of the animals also being inoculated intradermally into the right thigh.

#### *Tonsils*

**Figure 4:**
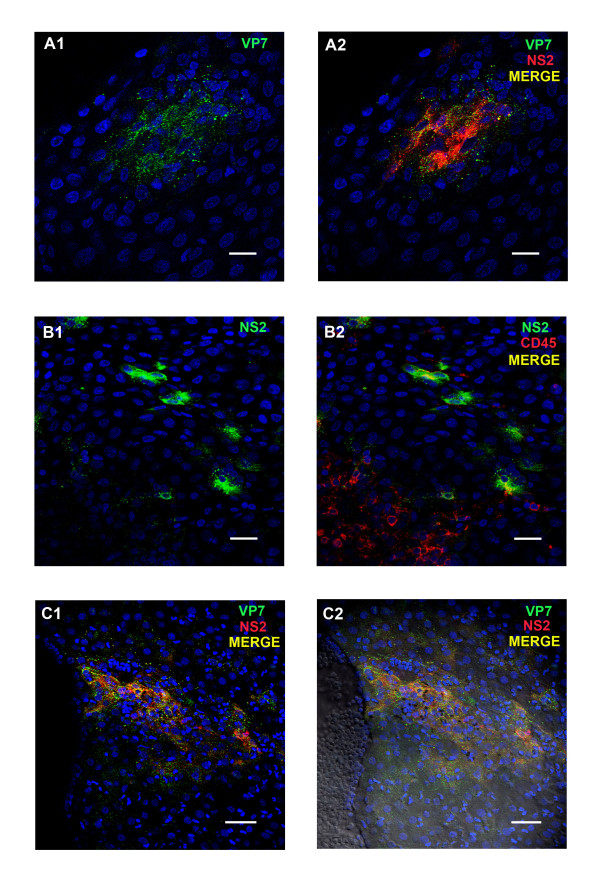
**BTV viral proteins in tissue sections of sheep tonsil at different time points pi with BTV-2.A1/A2:** BTV proteins VP7 (ORAB36-green) and NS2 (ORAB53- red) co-localised in a focal area of the tonsil’s epithelium at 6 dpi. Scale bar: 20 μm. **B1/B2:** BTV protein NS2 (ORAB53- green) in CD45^+^ positive cells (mAb151- red) in the epithelium of the tonsil at 4 dpi. Scale bar: 20 μm. **C1/C2:** BTV proteins VP7 (ORAB36- green) and NS2 (ORAB53- red) in the amygdaloid tissue of the tonsil at 6 dpi. Scale bar: 40 μm.

Although no viral proteins were detected in the tonsils at 3 dpi, BTV structural and non-structural protein expression were subsequently detected in the animals infected with BTV-2 (euthanized on 4 to 9 dpi) in three different areas of the tonsil. Firstly, both VP7 and NS2 were detected in patches of cells in the tonsil epithelium, in samples from the sheep euthanized at 4, 6 and 9 pi (Figure 4 a1/a2). These patches were not found in all tonsil sections, although these analyses were complicated by the partial or complete absence of the epithelium from some sections. Such patches of cells in the epithelium containing NS2 also showed co-localised staining with the anti-CD45 (leukocyte specific) antibody (Figure 4 b1/b2), indicating that intra-epithelial leukocytes were infected with BTV.

Secondly, the endothelial layer of the lymphatic ducts also contained large amounts of both structural and non-structural BTV proteins at 6 dpi, and viral proteins were also detected in the amygdaloid tissue close to lymphatic ducts of some tonsil sections (Figure 4 c1/c2).

Thirdly, at 6, 8 and 9 dpi viral proteins were found in the endothelial cells and lumen of the micro-vascular system, as well as in a few leukocytes.

#### *Lips*

**Figure 5:**
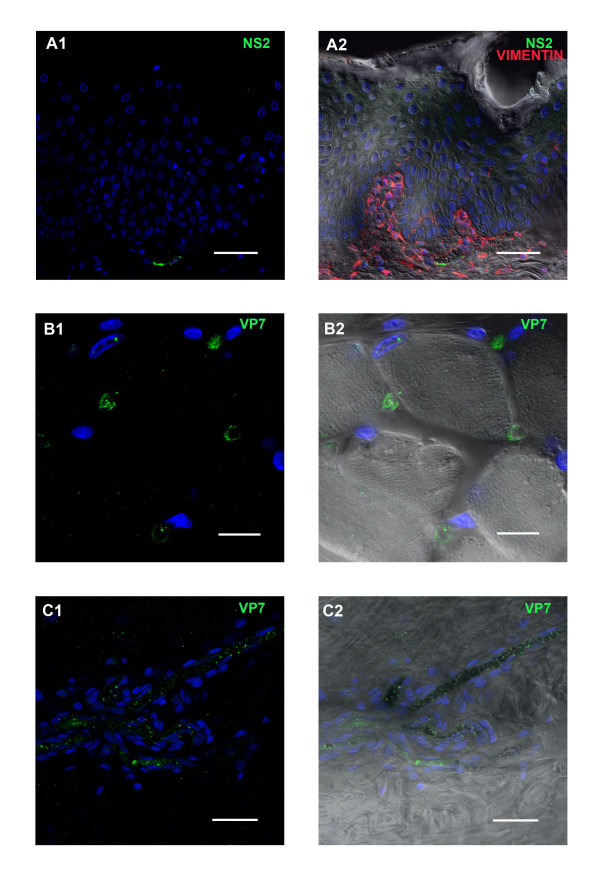
**BTV viral proteins in tissue sections of other sheep organs at different time points pi with BTV-2.A1/A2:** BTV protein NS2 (ORAB53- green) in the endothelium of a capillary in the dermis underneath the epithelium of the labia at 6 dpi. Vimentin, a member of the intermediate filament family commonly used as marker for mesodermally derived tissues, is shown in red to demonstrate the dermal layer in contrast to the epithelium (which does not contain vimentin). Scale bar: 40 μm. **B1/B2:** BTV protein VP7 (ORAB36-green) in the endothelium of capillaries between muscle blocks of the tongue at 4 dpi. Scale bar: 20 μm. **C1/C2:** BTV protein VP7 (ORAB36-green) in the endothelium of small blood vessels in the dermis of the skin at 6 dpi. Scale bar: 40 μm.

Both VP7 and NS2 were detected in the dermis of the lips as early as 3 dpi. Capillaries immediately beneath the epithelium stained positively for both BTV proteins. Co-localisation of vimentin and NS2 (ORAB53) indicates BTV infection and active replication in either endothelial cells or fibroblasts. In the majority of cases the infected cellular structures that stained for viral proteins were clearly identified as capillaries by DIC (Figure 5 a1/a2). However, some of the infected cells were morphologically similar to fibroblasts but could not be further identified.

The number of infected capillaries detected throughout the dermis, increased from 6 to 8 dpi. Tissue sections from these sheep also contained viral proteins in the capillaries around skin glands (not shown). BTV infected leukocytes were rarely identified in the lips.

#### *Tongue*

Both NS2 and VP7 were detected in the capillaries between muscle blocks of the tongue from 3 dpi onward (Figure 5 b1/b2). However, no viral proteins were detected in any of the muscle cells or leukocytes. On 6 and 8 dpi, larger numbers of capillaries, and some of the larger blood vessels in the lamina propria, were infected, a few of which were directly beneath the epithelium.

#### *Spleen*

A few leukocytes and occasional endothelial cells that contained both structural and non-structural viral proteins, were detected in the spleen from 3 dpi onwards and the number of infected leukocytes increased by 2–3 times 6–9 dpi. More infected endothelial cells (about ~ 3 times as many) were also identified between 6–8 dpi, mostly within capillaries, but occasionally also within the larger blood vessels.

#### *Liver*

No viral proteins were detected in sections of the liver taken at 3 or 4 dpi. However, NS2 and VP7 were both detected in endothelial cells and the lumen of several micro-vascular vessels at 6 and 8 dpi (data not shown). No other infected cells, hepatic or leukocytes, were detected. The number of viral protein positive endothelial cells had decreased by 9 dpi and only single or few positive endothelial cells were detected in some but not all sections.

#### *Heart*

Only a few infected capillaries (containing both VP7 and NS2) were detected within the myocardium taken from the left ventricle (not shown) at 4, 6 and 8 dpi. while none could be detected on 3 and 9 dpi. No viral proteins were detected in the muscle cells or leukocytes.

#### *Lungs*

Lung tissues could not be analysed by confocal microscopy due to a high level of background staining.

#### *Skin*

**Figure 6:**
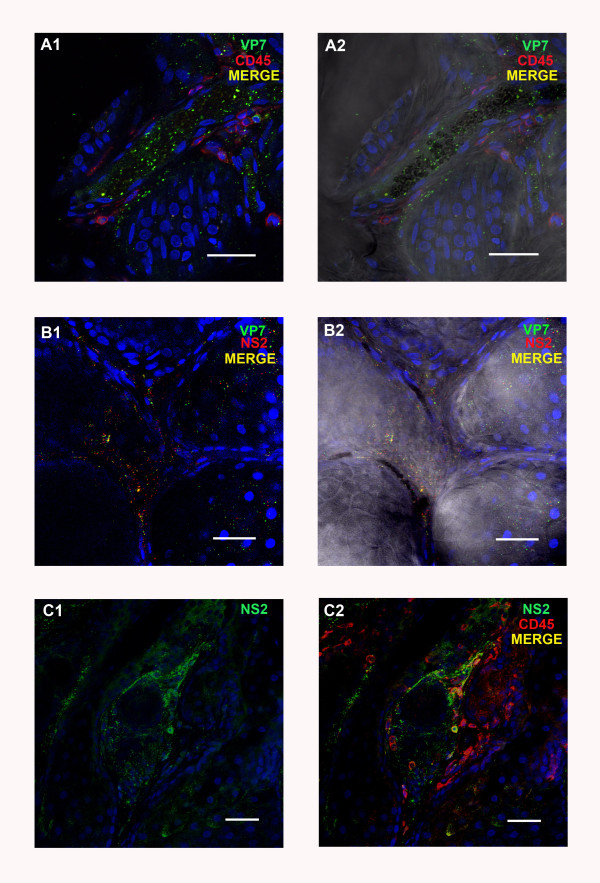
**BTV viral proteins in tissue sections of sheep skin at different time points pi with BTV-2.A1/A2:** BTV protein VP7 in the lumen and endothelium of a blood vessel of the skin at 6 dpi. Leukocytes (characterised as CD45^+^/mAb151-red) are also present around the blood vessel. Scale bar: 40 μm.** B1/B2:** BTV proteins NS2 (ORAB53-red) and VP7 (ORAB36-green) in capillaries around glands in the skin at 6 dpi. Scale bar: 40 μm. **C1/C2:** BTV protein NS2 (ORAB53- green) present around a gland and in leukocytes (CD45^+^/mAb151-red) of the skin at 6 dpi. Scale bar: 40 μm.

BTV proteins (VP7 and NS2) were detected from 3 dpi onwards, in endothelial cells of capillaries in the dermis. Both viral proteins (but especially VP7) were sometimes also detected either free in the lumen of these vessels or in close association with erythrocytes. Some vimentin positive cells, situated immediately beneath the epithelium, were also positively stained for both viral proteins. The number of infected capillaries had increased by about ~3-5 times, 6–9 dpi (Figure 5 c1/c2) and viral proteins were also detected in some of the larger blood vessels (Figure 6 a1/a2). Infected capillaries were particularly evident in the immediate vicinity of dermal glands (Figure 6 b1/b2). At 6 dpi large amounts of both viral proteins were detected around these glands (Figure 6 c1/c2). Leukocytes, identified by anti-CD45 Ab staining, were detected at 6, 8 and 9 dpi, around infected blood vessels, capillaries and skin glands and several of them were positive for BTV antigen (see Figure 6 a1/a2 and c1/c2). No differences were observed in the amount of BTV proteins present in skin sections from areas that had been exposed to the biting of *C. sonorensis,* as compared to non-exposed sites.

In summary most cells (endothelial cells and/or leukocytes) infected with BTV-2 detected by immuno-labelling and confocal microscopy were present in the mandibular and prescapular lymph nodes, the tonsil and the skin.

### Confocal microscopy and immuno-fluorescence of a sheep (8 dpi) and calf (10 dpi) infected with BTV-8 (NET2006/01)

**Figure 7:**
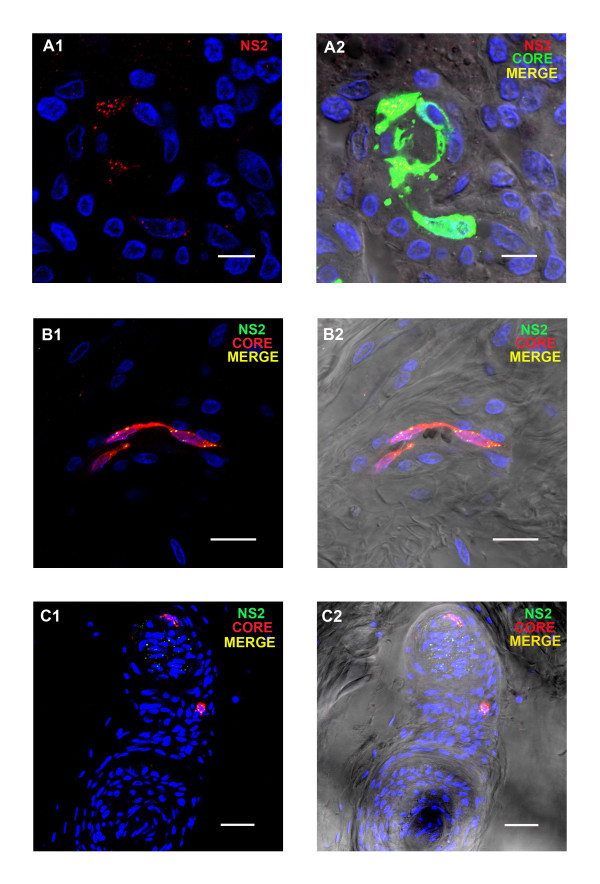
**BTV viral proteins in tissue sections of selected sheep organs a 8 dpi with BTV-8.A1/A2:** BTV core proteins (ORAB06- green) and NS2 (ORAB01- red) in the endothelium of a vessel in the prescapular lymph node at 8 dpi. Scale bar: 8 μm. **B1/B2:** BTV core protein (ORAB06- red) and NS2 (ORAB268) in the endothelial cells of a capillary in the skin at 8 dpi. Scale bar: 20 μm. **C1/C2:** BTV core proteins (ORAB06- red) and NS2 (ORAB268-green) in a gland branching off a hair in the skin at 8 dpi. Scale bar: 40 μm. The staining pattern of viral proteins in organs of infected sheep was comparable between animals infected with two different BTV strains, namely BTV2 and BTV 8, and between different antibodies to the same and/or additional viral proteins.

Sections of prescapular lymph nodes from the BTV-8 infected sheep contained large numbers of capillaries and larger blood vessels that were positive for viral proteins (core proteins and NS2-Figure 7 a1/a2). Other cellular components in the prescapular lymph node including lymphocytes also contained structural and/or non-structural BTV proteins. BTV proteins were detected in much smaller numbers of capillaries in the inguinal lymph node of the sheep and only very few BTV positive leucocytes or single capillaries were detected in sections of the sheep tonsil.

The prescapular lymph node of the infected calf only contained BTV protein in a few small capillaries (not shown), while no BTV proteins were detected in sections of the inguinal lymph node or the tonsil. Both animals stained positively for viral proteins (core, NS2 and VP7) in a few capillaries of the dermis of the tongue, sometimes close to the epithelium. Several infected capillaries which contained both NS2 and BTV core proteins were also identified in skin sections of the sheep. In most cases the viral proteins were associated with endothelial cells (Figure 7 b1/b2) but on a few occasions structural and non-structural viral proteins were also found in the skin glands themselves (Figure 7 c1/c2) as observed on 6 dpi in the BTV-2 infected animals (Figure 6 c1/c2). A few single virus protein positive capillaries were also identified in the skin of the infected calf (not shown). Overall the micro-vascular system in the skin of both BTV-8 infected animals contained a relatively high number of infected endothelial cells as compared to the other tissues examined. The number and pattern of BTV positive cells in tissue sections of the BTV-8 infected sheep at 8 dpi were comparable with tissue sections of the BTV-2 infected sheep at 8 dpi other than that BTV infected cells were not seen here in the epithelium or amygdaloid tissue of the tonsil.

## Discussion

The demonstration of BTV replication in endothelial cells of skin capillaries as well as in leukocytes in the skin is the most important observation of the study presented here. Previous studies have detected BTV or EHDV in the skin of infected ruminants, especially around hair follicles or in the papillae of the hair [12,13,17,34]. Nevertheless, the skin has not been considered to be an important “replication” organ for these viruses, partly because virus isolation from skin samples indicated only low or inconsistent virus loads [10,16,20]. This may at least partly reflect the fibrous and compact nature of ovine and bovine skin, making it difficult to disrupt and effectively homogenise samples for virus isolation or RNA extraction.

Here, we report the replication of BTV in endothelial cells of the skin capillaries as well as in leukocytes present in the skin, as early as 3 dpi. Infected skin glands, which morphologically resemble sebaceous glands and on a few occasions were directly associated with a hair follicle, were also detected. Haemorrhages were observed in skin from all over the body (Figure 1b), indicating that endothelial cells of dermal capillaries in the entire skin may become infected. Since the skin represents the largest single organ in the body this suggests that collectively it may also be a major site for BTV replication at least during the acute and early stages of BTV infection investigated in this study. However no difference in the presence and/or replication of BTV in the skin was detected between skin sections taken from *C. sonorensis* feeding sites and sections from normal, non-feeding sites. Hence the influence, if any, of *Culicoides* saliva proteins on BTV replication requires further investigation.

Immuno-labelling and confocal-microscopy were used to detect non-structural viral protein NS2 showing that BTV replicates primarily in two cell types within infected sheep, endothelial cells (mainly of small capillaries) and agranular mononuclear leukocytes. Morphologically the infected leukocytes resembled monocytes,/macrophages, lymphocytes and/or dendritic cells . Although BTV infected γδ T-cells (WC-1 positive cells) were detected in the skin of infected sheep, WC-1 negative leukocyte populations were also clearly infected (data not shown), indicating that several subsets of agranular leukocytes can become infected with BTV. Unfortunately the phenotype of these BTV infected leukocytes (other than γδ T cells) could not be determined as the fixation technique employed restricted the availability of useful antibodies against ovine cell surface markers.

The detection of BTV proteins in lymphatic organs and in the endothelium of blood vessels in the skin, even at the earliest time point investigated (3 dpi), suggests that replication in mononuclear leukocytes and vascular endothelial cells was simultaneous rather than consecutive. Additionally infected endothelial cells and/or leukocytes were not evenly distributed throughout infected organs but appeared to cluster in close proximity to each other suggesting foci of infection caused by local spread of the virus within the organ itself.

Although BTV infection causes a high level of viraemia in infected ruminants the level of replication in most infected organs appeared to be relatively low (as assessed by numbers of infected cells) when compared to similar studies of other viruses such as FMDV [28]. The high level of viraemia may reflect tropism of BTV for abundant cell types (endothelial cells) and/or widely distributed cell subsets (leukocytes). This contrasts with viruses that replicate to high levels in the cells of only a few organs or at a few specific replication sites, e.g. FMDV in the basal layer of the epithelium in the tongue, soft palate and coronary band [28] or rotavirus in the mature epithelial cells at the apices of small intestinal villi [35].

The lymph nodes of the head and the tonsils contained the largest numbers of BTV infected cells. Like the skin, tonsils have not previously been identified as a major site for BTV infection and/or replication [10,11,16,20]. In further contrast to other studies [36] we detected little virus replication in the spleen of infected animals. The differences in organ tropism that were observed here compared to previous studies may reflect differences between the infecting virus strains, between individual host breeds or even between individual animals. The limited number of infected cells that we detected in the spleen compared to earlier studies could also be a reflection of the different techniques employed and/or between virus replication and virus presence. The red pulpa of the spleen is a major blood reservoir and high virus levels detected in the spleen could simply reflect a high systemic viraemia. Later in infection BTV particles are mainly embedded in the cell membrane of erythrocytes and may therefore be hidden from antibodies [37,38] and so would remain undetected by confocal microscopy. Such particles could potentially be detected by either virus isolation or RT-PCR, techniques that do not distinguish between virus presence and replication. Further studies are needed to determine if the high level of virus commonly observed in the spleen of BTV infected ruminants is also associated with a high level of replication within this organ or due to virus particles present in the blood circulation.

Variation in endothelial cell susceptibility has been suggested as an explanation for differences in the clinical manifestation of BT in sheep and cattle [39,40]. Endothelial cells from different tissue origins within the mammalian host also exhibit differences in BTV or AHSV susceptibility and subsequent immune responses [15,41,42]. This suggests that variations in the susceptibility of vascular endothelial cells within different organs of the infected host could potentially determine tissue tropism and “organ-manifestation” of BTV, particularly the localisation of haemorrhages. Endothelial cells in the skin, lymph nodes of the head, tonsils and labia may therefore be more susceptible to infection by BTV-2 and 8, and were therefore detected as positive for virus replication at an early stage of infection. However, once a high level viraemia has been established, endothelial cells of less susceptible organs (such as heart, muscle, liver and kidney) may also become infected.

In general, the observed distribution of viral replication mirrored the damage observed either in micro- (histo-) or macro- (anatomical) pathological investigations. In each organ where petechial bleeding was observed infected endothelial cells were detected by immuno-fluorescence, suggesting that infection of endothelial cells may be at least partially responsible for the haemorrhages observed. However the extent of tissue damage seen in many organs did not seem to be reflected by the number of infected cells in these organs (endothelial cells and/or leucocytes). For many haemorrhagic fevers, including BT, it has been suggested that endothelial dysfunction may, at least in part, be attributable to indirect host immune mechanisms such as the release of vasoactive and/or proinflammatory mediators and/or coagulation disorders [15,43-45] . In this study the amount of viral antigen detected in the organs of infected sheep and the calf, at 9 or 10 dpi, was already lower than 6 and 8 dpi, suggesting a reduction of viral replication at these time points which is consistent with previous descriptions of BTV infection in ruminants [17,46]. Nonetheless severe clinical disease in sheep is often observed in the later stages of infection (> 9 dpi) when infectious viraemia is already subsiding [44]. Therefore it remains very likely that endothelial cell injury as a result of indirect inflammatory mechanisms rather than direct virus-mediated damage may have contributed to haemorrhagic lesions and tissue oedema observed in this study.

The only organ where histologically observed tissue damage did not reflect the level of anatomical damage and clinical dysfunction, was the lung. Despite being anatomically highly oedematous, only limited endothelial cell death and mild to moderate perivascular and peribronchal oedema were observed microscopically in the lungs. However, increased interstitial pressure can disperse oedema into nearby lower pressure areas (such as alveoli and body cavities) while the fluid is often lost during processing and thus grossly apparent pulmonary oedema may appear relatively mild or inconspicuous histologically. Alternatively, the above discussed indirect vascular leakage due to inflammatory host mechanisms could also play a role in causing the differences observed between the severe anatomical and clinical pulmonary oedema in the lungs and the limited endothelial cell damage detected histologically. In this study, high background staining, possibly caused by incomplete fixation or oedematous tissue damage, prevented the assessment of the relative level of virus replication in the lungs by confocal microscopy.

The current study was limited to only the acute and early stages of BTV infection (up to 10 dpi) in a relatively small number of animals (5 sheep in the longitudinal infection study, plus 2 animals [1 sheep, 1 calf] from a separate study). However, virus replication was clearly demonstrated in the skin of both BTV-8 infected animals, suggesting replication in the skin is a common aspect of BTV infections.

The observation of BTV replication in the skin raises questions concerning the validity of systemic viraemia determined from blood samples taken from the V. jugularis as a measure of virus levels in the periphery of the skin and therefore the amount of virus blood-feeding *Culicoides spp.* could potentially take up. *Culicoides* are “pool” feeders and do not take blood directly from blood vessels within the dermis (which are usually too deep for their proboscis to reach). Instead they lacerate the skin to feed on the effusion into this injury, which includes blood, skin cells and lymph [47]. The replication of BTV in the skin therefore provides a local source of infectious virus for vector insects in addition to virus present in the skin capillary system as a result of systemic viraemia. *Culicoides* can also use the hair as a guide and route of access to the dermal layer of the skin often, feeding immediately alongside the hair shaft itself (P.S. Mellor personal communication) suggesting that infection of capillaries around perifollicular glands and of the glands themselves may also be directly important for infection of these vectors. Further research will be required to investigate if infected ruminants with a relatively low level systemic viraemia could still represent a transmission risk to individual adult *Culicoides* feeding directly on skin areas where the virus is replicating, especially as viral antigens have been detected in the skin as early as 2–3 dpi here and in other studies [34].

## Competing interests

The authors declare that they have no competing interests.

## Authors’ contributions

KD designed the experimental protocols, coordinated and conducted the in vivo work, carried out immunolabelling and confocal microscopy, analysis of data and wrote the manuscript, PM advised on the study design, supervised and carried out confocal microscopy, assisted with the analysis of data and the manuscript, JS carried out in vivo work and sample preparation, immunolabelling and confocal microscopy, SA, EV and HE carried out in vivo work and contributed to the data analysis and the manuscript, HWB carried out the histological examination and assisted with the analysis of data and the manuscript, JB contributed to the experimental design and analysis of data, HHT, PSM and PPCM initiated the study, advised and supervised the design, the analysis and interpretation of data and assisted with writing the manuscript. All authors have read and approved the final manuscript.
